# An Identity-Affirming Web Application to Help Sexual and Gender Minority Youth Cope With Minority Stress: Pilot Randomized Controlled Trial

**DOI:** 10.2196/39094

**Published:** 2022-08-01

**Authors:** Jose Bauermeister, Seul Ki Choi, Emma Bruehlman-Senecal, Jesse Golinkoff, Arianna Taboada, Joshua Lavra, Lionel Ramazzini, Fred Dillon, Jana Haritatos

**Affiliations:** 1 Department of Family and Community Health University of Pennsylvania Philadelphia, PA United States; 2 Hopelab San Francisco, CA United States

**Keywords:** lesbian, gay, bisexual, transgender, queer, and other sexual and gender minority, LGBTQ+, youth, adolescence, discrimination, minority stress, mental health, resilience, sexual and gender minority, SGM, intersectionality

## Abstract

**Background:**

Efficacious mental health interventions for sexual and gender minority youth have had limited reach, given their delivery as time-intensive, in-person sessions. Internet-based interventions may facilitate reach to sexual and gender minority youth; however, there is little research examining their efficacy.

**Objective:**

This study aims to describe the results of a pilot randomized controlled trial of *imi*, a web application designed to improve mental health by supporting lesbian, gay, bisexual, transgender, queer, and other sexual and gender minority identity affirmation, coping self-efficacy, and coping skill practice.

**Methods:**

Sexual and gender minority youth (N=270) aged 13 to 19 (mean 16.5, SD 1.5) years and living in the United States were recruited through Instagram advertisements. Approximately 78% (210/270) of the sample identified as racial or ethnic minorities. Participants were randomized in a 1:1 fashion to the full *imi* intervention web application (treatment; 135/270, 50%) or a resource page–only version of the *imi* site (control; 135/270, 50%). The *imi* application covered four topical areas: gender identity; lesbian, gay, bisexual, transgender, queer, and other sexual and gender minority identity; stress and coping; and internalized homophobia and transphobia. Participants explored these areas by engaging with informational resources, exercises, and peer stories at a self-guided pace. Both arms were assessed via web-based surveys at baseline and 4-week follow-up for intervention satisfaction, stress appraisals (ie, challenge, threat, and resource), coping skills (ie, instrumental support, positive reframing, and planning), and mental health symptoms among other outcomes. Main *intent-to-treat* analyses compared the arms at week 4, controlling for baseline values on each outcome.

**Results:**

Survey retention was 90.4% (244/270) at week 4. Participants in the treatment arm reported greater satisfaction with the intervention than participants in the control arm (t_241_=–2.98; *P*=.003). The treatment arm showed significantly greater improvement in challenge appraisals (ie, belief in one’s coping abilities) than the control (Cohen *d=*0.26; *P*=.008). There were no differences between the arms for threat (*d*=0.10; *P*=.37) or resource (*d*=0.15; *P*=.14) appraisals. The treatment arm showed greater increases in coping skills than the control arm (instrumental support: *d*=0.24, *P*=.005; positive reframing: *d*=0.27, *P*=.02; planning: *d*=0.26, *P*=.02). Mental health symptoms improved across both the treatment and control arms; however, there were no differences between arms. Within the treatment arm, higher engagement with *imi* (≥5 sessions, >10 minutes, or >10 pages) predicted greater improvement in stress appraisals (all *P* values <.05).

**Conclusions:**

The results provide initial evidence that asynchronous psychosocial interventions delivered via a web application to sexual and gender minority youth can support their ability to cope with minority stress. Further research is needed to examine the long-term effects of the *imi* application.

**Trial Registration:**

ClinicalTrials.gov NCT05061966; https://clinicaltrials.gov/ct2/show/NCT05061966

## Introduction

### Background

Compared with their cisgender, heterosexual peers, sexual and gender minority (SGM) youth are at increased risk of experiencing a wide variety of negative mental health outcomes [1]. In a recent national surveillance survey conducted by the Centers for Disease Control and Prevention, 55% of gay, lesbian, or bisexual youth reported poor mental health during the prior 30 days. SGM youth were also twice as likely as their non–SGM youth counterparts to report feeling sad or hopeless and nearly 3 times as likely to have considered attempting suicide [2]. These disparities may vary further by race and ethnicity. A 2021 national survey of SGM youth (aged 13-24 years) in the United States sponsored by the Trevor Project found that racial and ethnic minority SGM youth were more likely than White non-Hispanic SGM youth to have seriously considered suicide in the prior year [3]. Taken together, these data underscore the need to address the well-being of diverse SGM youth populations through innovative mental health interventions [4].

The minority stress model [5,6] provides a framework for understanding the higher prevalence of psychological distress and negative mental health outcomes for SGM youth, as well as for identifying interventions to improve SGM individuals’ mental health [7]. The minority stress theory proposes that SGM health disparities can be explained in large part by discrimination from a hostile homophobic and transphobic culture, which creates stressors unique to minority identity [8]. These stressors include harassment, victimization, internalized homophobia and transphobia, and expectations of rejection. These disparities may be further compounded if individuals experience multiple minority stressors because of having >1 minority identity (eg, discrimination because of sexuality, gender, racial, and ethnic identity) [9-12]. For instance, racial and ethnic minority SGM youth may experience sexual or gender minority stress within their racial and ethnic communities while also experiencing racial and ethnic minority stress within lesbian, gay, bisexual, transgender, queer, and other SGM (LGBTQ+) communities.

The transactional model of stress and coping [13] notes that individuals’ ability to respond to stress and reduce its impact on their well-being begins with an assessment of the stressor (ie, primary appraisal) and their confidence and ability to respond to the stressor (ie, secondary appraisal). Interventions designed to target and transform appraisals of stress from that of a threat to more of a challenge through cognitive and behavioral coping strategies have been shown to support the mental health and well-being of adolescents [14-16]. Efficacious mental health interventions for SGM youth have focused on providing resources that scaffold the ability of SGM youth to perceive minority stressors as a challenge to be faced and overcome rather than as a threat, including strengthening the coping skills of SGM youth, affirming SGM identities, and strengthening supportive social connections [17-19].

Although prior research suggests that face-to-face interventions that include these components may improve the mental health of SGM youth [20], the reach and scalability of these programs have been challenging, given their time intensity and need for synchronous interactions, which have become increasingly difficult to coordinate amidst the COVID-19 pandemic [21,22]. At the same time, the need for scalable mental health resources has become particularly acute in recent years. For example, data from a large US survey of teenagers conducted by Common Sense Media [23] indicated that the amount of time SGM youth spent searching for mental health information on the web substantially increased during the pandemic.

In recent years, technology-assisted interventions have been posited to help decrease implementation challenges by serving as supplemental strategies to face-to-face psychotherapy. For example, Lucassen et al [24] found that their modular computer-delivered cognitive behavioral therapy program was feasible, acceptable, and effective in their pilot study with 21 sexual minority adolescents, aged 13 and 19 years, in New Zealand. Other programs have sought to use web-based interventions to circumvent barriers to accessing affirming in-person services. For example, Craig et al [18] found that SGM youth in Canada (N=46; age 14-29 years) who participated in their 8-session, manualized, and synchronous pilot telehealth group intervention found the program to be acceptable. Although their design did not allow for randomization, preliminary efficacy analyses noted improvements in stress appraisals, cognitive and behavioral coping skills, and depressive symptomatology in the web-based group program when compared with youth in the wait-list control group. Taken together, these findings are promising and highlight technology’s potential as a modality to deliver mental health interventions for SGM youth.

To date, there is limited research examining the efficacy of web-based platforms for helping SGM youth cope with minority stress asynchronously. For instance, in a pilot randomized trial, Schwinn et al [25] found that their tailored, 3-session web-based intervention resulted in decreases in perceived stress and increases in coping and problem-solving skills when compared with youth in the control arm at a 3-month follow-up. Egan et al [26] designed an innovative, web-accessible role-playing game intervention. Although they found high acceptability for their program among SGM youth participating in their randomized trial, they did not observe any improvements at the 1- or 2-month follow-up across coping skills, depression and anxiety symptoms, or knowledge and use of web resources.

### Study Objectives

Although these findings suggest that SGM youth perceive web-based asynchronous interventions to be acceptable, there is a need to increase the empirical evidence base for the efficacy of these interventions [27]. Moreover, the generalizability of the aforementioned findings to racial and ethnic minority populations has been constrained, given the limited representation of these groups in prior research. Given the current state of the science, this study sought to test the acceptability and preliminary efficacy of an asynchronous web application—*imi*—among a predominantly racial and ethnic minority sample of SGM youth aged between 13 and 19 years living in the United States. The *imi* application was designed to facilitate SGM identity affirmation, promote a sense of connectedness to the LGBTQ+ community, and encourage cognitive and behavioral coping skill practice. In partnership with a racially and ethnically diverse group of SGM youth, we co-designed the *imi* application to be directly responsive to their needs by leveraging the visual, aesthetic, and interactive capacities of a web-based interface to deliver identity-affirming experiences that could support the intervention’s engagement and efficacy.

Our study had 4 main objectives. First, we examined the acceptability of the *imi* application in a diverse sample of 270 SGM youth. Given our use of human-centered design principles and the involvement of SGM youth in the design of the *imi* application, we expected that participants randomized to receive the *imi* application would report greater acceptability and satisfaction than participants assigned to a resource-only version of the *imi* application (the control arm), which did not contain any of the newly created interactive coping and identity-affirming content designed with LGBTQ+ youth. Second, we examined the preliminary efficacy of the *imi* application as a digital tool for increasing adaptive stress appraisals among SGM youth (primary outcome). Given the *imi* application’s focus on teaching cognitive and behavioral coping skills, we hypothesized that participants assigned to receive the *imi* application would be more likely to appraise stress as a surmountable challenge and less likely to appraise stress as threatening by the 4-week follow-up relative to the control arm. Third, we examined the preliminary efficacy of the *imi* application across five secondary outcomes related to the mental health of SGM youth: cognitive and behavioral coping skills, identity affirmation and connectedness to the LGBTQ+ community, internalization of blame for minority stress, sense of belonging, and anxiety and depression symptoms. We predicted that the *imi* application would be more likely to improve SGM youth’s outcomes across these domains relative to the control arm. Finally, as exploratory analyses, we examined participants’ engagement with the *imi* application relative to the control arm. We also explored whether participant engagement with the *imi* application (ie, counts of user sessions, time spent, and the number of pages visited) predicted improvement in primary and secondary outcomes.

## Methods

### Study Design

This pilot randomized controlled trial evaluated the acceptability and initial efficacy of the *imi* application at the end of the 4-week active study period. Participants were randomly assigned in a 1:1 fashion to receive either the *imi* application (treatment arm) or a resource page–only version of the *imi* site called “asterix,” which linked out to a series of LGBTQ+-specific external mental health resources (resource-only control arm). We collected survey data via web-based self-completed Qualtrics surveys administered at baseline and at a 4-week follow-up.

### Ethics Approval

The University of Pennsylvania Institutional Review Board approved all study procedures (protocol 849509), and the study was registered on ClinicalTrials.gov (NCT05061966). A waiver of parental consent was granted to ensure that youth who might not yet be out to their parents or have less parental support, and thus could benefit from an identity-affirming tool, could participate in the study.

### Participants and Recruitment

Participants were SGM youth recruited through Instagram between October and November 2021. To be eligible for this study, the youth had to (1) be aged 13 to 19 years (inclusive); (2) identify as a sexual or gender minority; (3) reside within the United States; (4) have English literacy; (5) have access to a device with internet access, a web browser, and SMS text messaging capabilities; and (6) be willing to participate in study activities for 4 weeks.

A target sample size of 250 participants was selected to allow for the detection of arm differences in week 4 outcomes, which were medium to small in size or larger (Cohen *d*≥0.35) after accounting for the potential loss of participants because of attrition or noncompliance. These effect sizes are consistent with those observed in previous research on digital mental health tools for SGM youth [18,25].

### Study Procedures

All study activities were conducted remotely, and web-based screening and survey assessments were delivered through the Qualtrics software. Prospective participants clicked on a paid advertisement and completed a brief screening survey. Individuals were emailed a link to the baseline survey, which contained the informed consent form (Multimedia Appendix 1), and participants were given 2 weeks to complete the survey. The baseline survey contained 8 of the same or similar questions as were asked in the screener. Following the established best practices for participant verification [28-30], the staff compared each applicant’s screener and baseline data for these 8 questions, in addition to the metadata (eg, IP addresses registered in the United States, review of the time taken to complete the survey, and answers in hidden “honey pot” fields). The study staff also manually checked all screeners that met basic eligibility criteria to eliminate duplicate and fraudulent entries. If any significant inconsistencies were identified, applicants were emailed and asked to respond via email or phone to resolve the issue.

Once participants completed the baseline survey and passed the verification procedure, they were considered to be enrolled in the study. Enrolled participants were randomized in a 1:1 ratio into the *imi* (treatment; received full intervention content) or *asterix* (control; received pared-down resources-only version of the *imi* application) arm. Within 1 business day of completing the baseline survey, each participant was emailed a unique link to the *imi* or *asterix* web application. Participants were compensated with a US $30 Amazon e-gift card once they registered for an account on *imi* or *asterix*. The informed consent form encouraged participants to log into their digital resource at least twice a week and informed them that the study team would be able to track where they went within the web application, what features they used, what content they saw, and the duration of time they spent on the web application. Depending on their communication preferences, participants were emailed or texted every 7 days after enrollment to remind them to use the web application. On day 28, participants were sent a link to the follow-up survey, which they had 14 days to complete. Once completed, they received a US $40 Amazon e-gift card. Authors were not blind to participants’ conditions during data collection or analysis; however, as all intervention activities were self-guided and all outcome measures were self-assessed by participants, there was no interaction between study staff and participants that could have led to response biases on the part of participants because of demand characteristics. No adverse events were reported during the trial.

### Intervention Development Study Procedures

**Figure 1:**
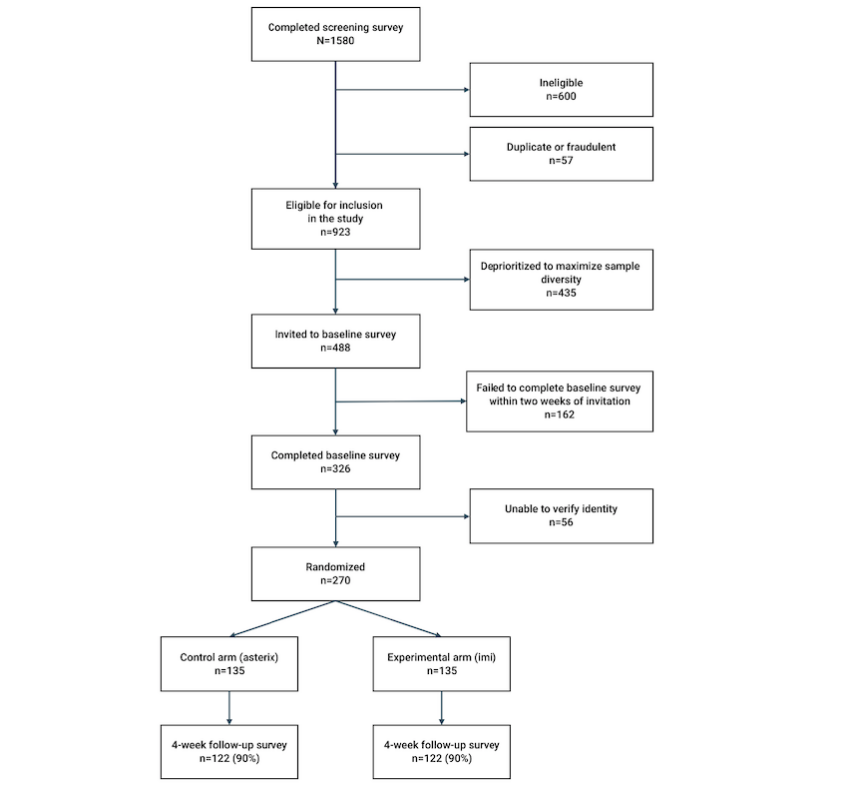
CONSORT (Consolidated Standards for Reporting Trials) diagram.

The *imi* application was built by Hopelab, a nonprofit social innovation laboratory, in collaboration with CenterLink, an international nonprofit organization and member-based association of LGBTQ+ centers serving their local and regional communities. Before launching the pilot trial, Hopelab conducted formative work through interviews, focus groups, co-design sessions, and surveys of SGM youth. The *imi* web application content and visual elements were tailored based on youth feedback and contributions. Screenshots containing example content are presented in Figure 1. The core intervention modules or “guides” in the *imi* application were designed based on a review of the existing efficacious minority stress interventions in the literature and honed through feedback with scientific advisors during a 6-month iterative design phase. This included extracting evidence-based exercises from the literature to support key psychosocial targets (eg, cognitive and behavioral coping skills and identity affirmation) that could be adapted to a digital platform and prototyping digital “mini-interventions” that would be further developed in the final *imi* application content.

### Intervention Description

#### Experimental Arm

*imi* is a web application designed to facilitate LGBTQ+ identity affirmation, promote a feeling of connectedness to the LGBTQ+ community, and encourage cognitive and behavioral coping skill practice (Multimedia Appendix 2). The name *imi* (pronounced *eye-me*) is a nod to the idea that no matter who you are, you are you (ie, “I’m me”). The logo, designed as an ambigram that can be read in many orientations, represents the belief that even as one changes and evolves, they are exactly as they are. The *imi* application delivers fully automated information and skill practice across guides covering four content areas: (1) gender identity exploration (the *gender* guide), (2) sexual orientation and broader LGBTQ+ identity exploration (the *queerness* guide), (3) stress and coping (the *stress* guide), and (4) internalized homophobia and transphobia (the *stigma* guide). Examples of each guide’s goals and sample activities are as follows:

1. The *gender* guide allows youth to explore and affirm their gender identity and expression. For example, one activity uses a chat interface that allows users to experiment with different names and pronouns that fit their gender identity.
2. The *queerness* guide encourages youth to examine their LGBTQ+ identity through an intersectional lens and reflect upon what *queerness* means to them. For instance, one activity provides examples of the ways other LGBTQ+ youth define their queerness and guides the user through the creation of their own definition.
3. The *stress* guide provides psychoeducation on LGBTQ+ sources of minority stress (eg, discrimination, prejudice, and microaggressions) and teaches cognitive and behavioral coping skills through activities such as guided breathing, positive reframing exercises, and social support resources. Users are encouraged to select coping skills that work for them most effectively.
4. The *stigma* guide explains how negative and stereotypical messages become internalized and encourages users to examine and challenge their own internalized homophobia and transphobia. For example, one activity scaffolds users in developing a personalized affirmation to help them combat negative self-talk.

Each of the four guides comprises four types of content: (1) learning segments, (2) activities, (3) community content, and (4) external resources. Learning segments provide information about LGBTQ+ relevant vocabulary (eg, pronouns and commonly used terms for sexual and gender identity), queer history, and psychoeducation on minority stress and internalized stigma. Activities include interactive exercises, such as chat interfaces, drawing activities, and guided relaxation and mindfulness practices. Community content includes video, audio, and written stories and images of LGBTQ+ youth. External resources connect youth to externally linked content designed for LGBTQ+ youth, such as the Trevor Project and the Gay, Lesbian, and Straight Education Network’s *coming out* guides.

#### Both Arms

Participants in both arms received access to resource webpages that linked to freely accessible, preexisting crisis and noncrisis resources. Crisis resources included the National Suicide Prevention Lifeline, as well as resources specific to LGBTQ+ youth, such as TrevorChat. The noncrisis resources included moderated social networking and web-based chat spaces (eg, TrevorSpace and Q Chat Space), local LGBTQ+ centers (eg, CenterLink’s center-finder tool), self-guided web resources (eg, The “It Gets Better Project”), databases of LGBTQ+-affirming therapists (eg, Gaylista), and a guide to free digital mental health tools (One Mind PsyberGuide). The resource section also contains *safer browsing tips*, which provide web-based privacy and safety advice specific to LGBTQ+ youth.

The control arm only received access to the resource webpages described previously and did not have access to any of the other content in the *imi* application*.* This pared-down, resources-only version of the *imi* site that was provided to the control group was named *asterix* This control allowed for a test of whether the learning, interactive, and community content of *imi* guides had benefits above and beyond any benefit that might be derived from simply having a curated, unified access point for existing, freely available web-based resources for SGM youth.

Participants in both arms were instructed to try to visit their respective web applications at least twice a week during the 4-week active trial period but could engage with the content available to them however they wished, in any order, at their discretion. On the basis of their preferences, participants received either weekly texts or emails reminding them to log into their web application.

### Measures

#### Primary Outcomes: Stress Appraisals

The Stress Appraisal Measure for Adolescents [15] captures stress appraisals across 3 dimensions (challenge, threat, and resources). The 3-item Challenge subscale assesses perceptions of stress as a surmountable challenge (Cronbach α=.67). The 7-item Threat subscale measures perceptions of stress as having lasting, negative repercussions (α=.83). The 3-item Resources subscale assesses the belief that one has the necessary internal and external resources to cope with stress (α=.81). Responses to all items are recorded on a 5-point scale (1=*strongly disagree* to 5=*strongly agree*). A mean score was computed for each subscale, with higher values indicating greater endorsement of each respective stress appraisal.

#### Secondary Outcomes

##### Cognitive and Behavioral Coping Skills

Participants’ use of specific cognitive and behavioral coping skills were measured with items adapted from the brief 2-item Coping Orientation to Problems Experienced (COPE) inventory by Carver [31], specifically the self-distraction (α=.46), active coping (α=.70), emotional support (α=.79), instrumental support (α=.78), venting (α=.66), positive reframing (α=.61), planning (α=.71), acceptance (α=.65), self-blame (α=.79), substance use (α=.96), and behavioral disengagement (α=.75) subscales. Instructions were modified such that participants indicated how they had been coping with stress in their lives over the past 2 weeks on a 4-point scale (1=“I haven’t been doing this at all” to 4=“I’ve been doing this a lot”). A mean score was computed for each subscale, with higher values indicating greater use of that respective strategy.

##### Positive LGBTQ+ Identity

Two 5-item subscales from the Lesbian, Gay, and Bisexual (LGB) Positive Identity Measure [32] were used to measure positive LGBTQ+ identity factors. The first subscale, the *authenticity subscale* (α=.87), measured comfort with one’s own LGBTQ+ identity (eg, “I am honest with myself about my LGBTQ+ identity”). The second subscale, the community subscale (α=.87), measured a sense of connection to the broader LGBTQ+ community (eg, “I feel supported by the LGBTQ+ community”). Items were modified from LGB to LGBTQ+ to be inclusive of a range of SGM identities. Participants responded on a 7-point scale (1=*strongly disagree* to 7=*strongly agree*), and a mean score was computed for each subscale, with higher scores indicating greater authenticity and connection, respectively.

##### Internalization of Blame for Minority Stress

The 5-item Coping with Discrimination Scale–Internalization subscale [33] assesses the tendency to blame oneself for instances of SGM-related discrimination (eg, “I tend to wonder if I did something to offend the others involved”). Participants responded on a 6-point scale (1=*never* to 6=*always*), and positively worded items were reverse coded. We computed a mean internalization score, where higher scores indicate greater internalization of blame for minority stress (α=.84).

##### Sense of Belonging

A 5-item version of the Thwarted Belongingness subscale of the Interpersonal Needs Questionnaire [34] was used to measure perceived belonging. Participants responded to the items (eg, “These days, I feel disconnected from other people.”) on a 7-point scale (1=*not at all true*” to 7=*very true for me*). Positively worded items were reverse coded. We computed a sum score ranging from 5 to 35, where higher scores indicate a lack of sense of belonging (α=.81).

##### Anxiety and Depression Symptoms

The 7-item General Anxiety Disorder Scale [35] was used to examine symptoms of anxiety (eg, “Feeling nervous, anxious, or on edge”), and the 8-item Patient Health Questionnaire [36] was used to assess depressive symptoms (eg, “Feeling tired or having little energy”). These brief clinical measures have been used to screen for generalized anxiety and depression across a wide range of populations, including adolescents [37-39]. For both measures, participants rated the frequency of their symptoms over the past 2 weeks using a 4-point scale (0=*not at all* to 3=*nearly every day*). Items were summed to compute total scores for each construct, with higher scores indicating greater symptoms of anxiety and depression, respectively (General Anxiety Disorder-7 α=.88; Patient Health Questionnaire-8 α=.83).

#### Intervention Acceptability and Satisfaction

Participants rated the acceptability and their satisfaction with the *imi* and *asterix* applications at the 4-week follow-up. A modified version of the LGBTQ Appropriateness Scale [40] was used to assess the perceived relevancy and appropriateness of the web resources to SGM youth (eg, “This product appears to be relevant to people who identify as LGBTQ+”) using a 7-point scale (1=*strongly disagree* to 7=*strongly agree*). These questions were tailored for each web application’s features; we asked 12 items for the treatment arm (α=.91) and 9 items for the control arm (α=.86). Items were averaged, with a higher score indicating greater perceived appropriateness.

A measure of intervention satisfaction and suggestions for improvement created for this study were also included. The measure comprises a multiple-choice question (eg, “How would you rate your overall experience of this product?”; 1=*very bad* to 7=*excellent*) that was analyzed as a continuous variable and free-text responses (eg, “How could this product be more helpful to you?” [text box]).

A net promoter score (NPS; eg, “How likely would you be to recommend [*imi/asterix*] to a LGBTQ+ friend?”) was administered to further assess the perceived value of the interventions. Respondents answered on an 11-point scale (0=*not at all likely* to 10=*extremely likely*). Following established industry conventions for NPS, responses were recoded such that respondents who selected 9 or 10 were categorized as “Promoters,” those selecting 7 or 8 as “Passives,” and all others as “Detractors.”

#### Web Application Engagement

Participants’ actions in the *imi* and *asterix* applications were collected as paradata over the trial period. Each participant was provided with a unique link to their respective intervention. This link embedded participants’ study IDs as metadata within their individual accounts, allowing us to track how much time each participant spent in their web application, which pages they viewed, and which links they clicked. These paradata were transformed to characterize the amount, frequency, duration, and depth of engagement with the web-based intervention [41].

In this study, we derived four paradata metrics: (1) counts of user sessions, (2) time spent on each intervention, (3) the number of pages visited, and (4) external links clicked. Sessions were counted whenever there was a period of activity within the app, with a participant having the same session ID until they had a period of ≥15 minutes or more of inactivity with the application. We derived the variable *number of sessions* by counting the number of discrete sessions in which the participant engaged during the 4-week active trial period.

To measure time spent on the intervention, we tracked the number of minutes and seconds participants spent logged into their respective web resources during the active trial period. Each time a participant visited a page within the app, there was a record of their activity. We derived the variable *number of unique pages viewed* by summing the number of distinct pages a participant visited during the active trial period. Finally, each time a participant clicked on a link, a unique record of the click was generated. We summed the number of unique external links a participant clicked on during the active trial period to derive this value.

### Analytic Strategy

#### Overview

Descriptive analyses were conducted to summarize demographic characteristics among the study arms at baseline. Preliminary analyses tested baseline equivalence between the study arms on demographics, assessment of attrition, and differential attrition by study arm. We used SAS (version 9.4; SAS Institute) to conduct all analyses.

#### Intervention Acceptability

To test the interventions’ acceptability, we compared participants’ ratings of the 2 web resources (eg, intervention satisfaction and NPSs) using chi-square tests for categorical variables and Student *t* tests for continuous variables. Within the treatment arm, open-ended feedback was coded by 2 coders using rapid qualitative analysis methods [42]. Core questions guiding the coding included “What did you find most helpful about *imi*?” and “If you could change anything about *imi* or the guides in it, what would you change and why?” Quotes were chosen to illustrate salient themes.

#### Preliminary Efficacy

Analyses of all primary and secondary outcome variables were performed using an intention-to-treat approach, which included all available data from participants randomly assigned to the 2 arms, regardless of whether participants created an account within their respective web resources. Our primary efficacy analysis sought to examine whether there were differences in our primary and secondary outcomes between the 2 arms. We used linear regression to test the main effect of arm (treatment=1 vs control=0) on week 4 outcomes, adjusting for the baseline value of each respective outcome as a covariate. Recognizing that we were testing 2 versions of a web application (the resource-only version of *imi* called *asterix* and the full interactive *imi* intervention), we also tested for changes over time within each web application. For these within-arm analyses, we examined the mean changes from baseline to follow-up using paired *t* tests.

#### Engagement

Given the absence of standardized and generalizable threshold indicators to suggest adequate engagement across digital health interventions, we adopted an exploratory approach to the analysis of these data and created thresholds to define participants’ engagement with the intervention. After examining the distribution of the engagement data, we selected the following to define thresholds of use: ≥4 sessions, ≥10 minutes, ≥10 unique pages viewed, and ≥1 external link clicked. Given the high bivariate correlations between these indicators of engagement (Spearman ρ>0.65), as well as the exploratory nature of these analyses, we compared each engagement indicator separately by study arm using chi-square tests. Of note, treating the nonnormal data as continuous (with and without transformation of these data) yielded similar results.

Finally, we explored whether reaching these thresholds of use within the *imi* arm predicted differential gains across primary and secondary outcomes. We focused these analyses on three indicators capturing participants’ engagement within *imi*: time spent on site, number of sessions completed, and number of pages viewed, which captured the depth of participant engagement with that content which was unique to the treatment arm. We ran separate regression models because of the high multicollinearity among engagement metrics and the importance of examining the different scopes of paradata (eg, the amount, frequency, duration, and depth of engagement)*.* All models accounted for the baseline value of each respective outcome as a covariate.

## Results

### Screening and Enrollment

Of the 1580 individuals who completed the screening survey, 923 (58.4%) met all inclusion criteria and passed the duplicate and fraudulent entry checks (Multimedia Appendix 3). From this pool of 923 eligible participants, racial, ethnic, and gender minority youth were selectively invited to access the baseline survey to achieve a diverse participant pool.

We invited 488 participants in total to complete the baseline survey to ensure that the target enrollment of 250 participants would be reached, with the expectation that not all participants who expressed interest in participating would respond to further outreach or pass identity verification checks.

Of the 488 participants who were invited to complete the baseline survey, 162 (33.2%) failed to do so within the 2-week window, and another 56 (11.5%) participants did not pass the participant verification procedure (ie, there were discrepancies between information entered on screener and baseline surveys); thus, in total, 270 (55.3%) participants completed the baseline survey and were enrolled in the study.

### Sample Characteristics

Participants had a mean age of 16.49 (SD 1.49) years. Most of the participants resided in a metropolitan area, with the majority residing in the Southern or Western regions of the United States. A large proportion of participants identified with multiple races, gender identities, and sexual orientations. To represent the diversity and heterogeneity of the sample, we report these variables in a nonmutually exclusive fashion in Table 1. The sample was racially and ethnically diverse, with 77.8% (210/270) of participants identifying as racial or ethnic minorities. Similarly, 41.9% (113/270) and 39.6% (107/270) of the sample identified with multiple gender identities and sexual orientations, respectively. Nonbinary (94/270, 34.8%) and bisexual (96/270, 35.6%) were the response options selected most frequently. Participants expressed various levels of outness about their sexual orientation, with 26.3% (71/270) of participants noting that they were completely or mostly in the closet, whereas 39.3% (106/270) were mostly or fully out.

**Table 1:** Sociodemographic characteristics by study arm (N=270).

Characteristics		All (N=270)	Control (n=135)	Intervention (n=135)
Age (years), mean (SD)		16.49 (1.49)	16.42 (1.51)	16.56 (1.46)
**Geographic region, n (%)**				
	Metropolitan	249 (92.2)	126 (93.3)	123 (91.1)
	Micropolitan	14 (5.2)	7 (5.2)	7 (5.2)
	Small town	4 (1.5)	1 (0.7)	3 (2.2)
	Rural areas	3 (1.1)	1 (0.7)	2 (1.5)
**Census region, n (%)**				
	Northeast	37 (13.7)	16 (11.9)	21 (15.6)
	Midwest	53 (19.6)	29 (21.5)	24 (17.8)
	South	93 (34.4)	46 (34.1)	47 (34.8)
	West	87 (32.2)	44 (32.6)	43 (31.9)
**Education^a^, n (%)**				
	Kindergarten to 5th grade	3 (1.1)	2 (1.5)	1 (0.7)
	6th to 8th grade	98 (36.3)	48 (35.6)	50 (37)
	9th to 11th grade	100 (37)	51 (37.8)	49 (36.3)
	High school diploma or equivalent	52 (19.3)	26 (19.3)	26 (19.3)
	Some postsecondary education	17 (6.3)	8 (5.9)	9 (6.7)
**Subjective SES^b^, n (%)**				
	Wealthy	1 (0.4)	1 (0.7)	0 (0)
	Upper-middle class	43 (15.9)	18 (13.3)	25 (18.5)
	Middle class	119 (44.1)	57 (42.2)	62 (45.9)
	Working class	61 (22.6)	33 (24.4)	28 (20.7)
	Low income or poor	31 (11.5)	19 (14.1)	12 (8.9)
	I prefer not to respond	15 (5.6)	7 (5.2)	8 (5.9)
**Sex at birth, n (%)**				
	Male	61 (22.6)	32 (23.7)	29 (21.5)
	Female	209 (77.4)	103 (76.3)	106 (78.5)
**Living status, n (%)**				
	Living with parent, parents, guardian, or guardians	222 (82.2)	109 (80.7)	113 (83.7)
	Other	48 (17.8)	26 (19.3)	22 (16.3)
**Race and ethnicity^c^ (total count), n (%)**				
	American Indian or Alaska Native	14 (5.2)	10 (7.4)	4 (3)
	Asian	68 (25.2)	33 (24.4)	35 (25.9)
	Black or African American	64 (23.7)	29 (21.5)	35 (25.9)
	Hispanic or Latinx	73 (27)	42 (31.1)	31 (23)
	Middle Eastern or North African	9 (3.3)	7 (5.2)	2 (1.5)
	Native Hawaiian or other Pacific Islander	2 (0.7)	1 (0.7)	1 (0.7)
	White or Caucasian	130 (48.2)	64 (47.4)	66 (48.9)
	Other	9 (3.3)	4 (3)	5 (3.7)
**Racial or ethnic minority, n (%)**				
	Exclusive identifying as non-Hispanic White	60 (22.2)	28 (20.7)	32 (23.7)
	Identifying as racial or ethnic minority	210 (77.8)	107 (79.3)	103 (76.3)
**Gender identity^c^ (total count), n (%)**				
	Agender	6 (2.2)	2 (1.5)	4 (3)
	Cisgender man	28 (10.4)	15 (11.1)	13 (9.6)
	Cisgender woman	43 (15.9)	25 (18.5)	18 (13.3)
	Genderqueer	44 (16.3)	22 (16.3)	22 (16.3)
	Man	41 (15.2)	13 (9.6)	28 (20.7)
	Woman	41 (15.2)	21 (15.6)	20 (14.8)
	Nonbinary	94 (34.8)	51 (38.8)	43 (31.9)
	Transgender man	50 (18.5)	21 (15.7)	29 (21.5)
	Transgender woman	8 (3.0)	5 (3.7)	3 (2.2)
	Other	30 (11.1)	13 (9.6)	17 (12.6)
**Gender identity, n (%)**				
	Not questioning	218 (80.7)	107 (79.3)	111 (82.2)
	Questioning	52 (19.3)	28 (20.7)	24 (17.8)
**Multiple gender identities, n (%)**				
	Multiple identities	113 (41.9)	56 (41.5)	57 (42.2)
	Single identity	157 (58.1)	79 (58.5)	78 (57.8)
**Sexual orientation^c^ (total count), n (%)**				
	Asexual	42 (15.6)	16 (11.9)	26 (19.3)
	Bisexual	96 (35.6)	48 (35.6)	48 (35.6)
	Gay	47 (17.4)	27 (20)	20 (14.8)
	Lesbian	47 (17.4)	23 (17)	24 (17.8)
	Pansexual	40 (14.8)	21 (15.6)	19 (14.1)
	Queer	73 (27)	40 (29.6)	33 (24.4)
	Straight or heterosexual	4 (1.5)	1 (0.7)	3 (2.2)
	Other	21 (7.8)	8 (5.9)	13 (9.6)
**Sexual orientation, n (%)**				
	Not questioning	225 (83.3)	110 (81.5)	115 (85.2)
	Questioning	45 (16.7)	25 (18.5)	20 (14.8)
**Multiple sexual orientations, n (%)**				
	Multiple identities	107 (39.6)	54 (40)	53 (39.3)
	Single identity	163 (60.4)	81 (60)	82 (60.7)
**Outness, n (%)**				
	Definitely in the closet	24 (8.9)	12 (8.9)	12 (8.9)
	In the closet most of the time	47 (17.4)	19 (14.1)	28 (20.7)
	Half in the closet, half out of the closet	93 (34.4)	51 (37.8)	42 (31.1)
	Out of the closet most of the time	76 (28.2)	33 (24.4)	43 (31.9)
	Completely out of the closet	30 (11.1)	20 (14.8)	10 (7.4)

^a^The highest level of education completed.

^b^SES: socioeconomic status.

^c^Nonmutually exclusive categories; participants were allowed to select all that apply.

### Baseline Equivalence, Attrition, and Differential Attrition

Randomization resulted in baseline equivalence between the treatment and control arms on all demographics, primary and secondary outcomes. Our survey retention rate at the 4-week follow-up was 90.4% (244/270). In attrition analyses, comparing those who completed the follow-up survey (244/270, 90.4%) with those who did not (26/270, 9.6%), we found no significant condition differences in attrition linked to demographic characteristics or baseline scores on primary or secondary outcomes. Collapsing across the arms, participants who did not complete the follow-up survey were more likely to be younger (mean 15.42, SD 1.53 years vs mean 16.60, SD 1.44 years; 2-tailed t_268_=–3.95; *P*<.001) and reported fewer cognitive and behavioral coping skills at baseline (Multimedia Appendix 4; all *P* values <.01).

### Intervention Acceptability

Participants perceived the content of the intervention as appropriate to LGBTQ+ individuals, both in the treatment (mean 6.01, SD 0.85) and control arm (mean 5.85, SD 0.83; t_241_=–1.45; *P*=.15). Participants in the treatment arm rated their overall experience with the *imi* application (mean 6.01, SD 1.06) more positively than participants in the control arm (mean 5.59, SD 1.14; t_241_=–2.98; *P*=.003) and were also more likely to report that they would recommend the *imi* application to a friend (“Detractors” treatment: 26/121, 21.5%; “Detractors” control: 45/121, 37.2%; *χ*^2^_2_=8.9; *P*=.01).

When asked what they liked and found most useful about the *imi* application, participants remarked on the benefits of hearing the stories and seeing the images of other LGBTQ+ youth:

I liked being able to read other queer folks’ stories—no matter how many friends I have, it’s always nice to hear about other people with experiences or identities similar to my own and learn from what they’ve done.

Additional themes included being taught concrete strategies for managing stress and engaging with the activities (eg, the interactive chats and questionnaires) that encouraged identity exploration:

It gave lots of suggestions for stress relief, so I could focus on tackling stress myself. I don’t like turning to other people for help that much, so the self-aspect of it was helpful.

I really liked the activities—especially the one where I got to test out a new name. It made me feel seen.

When asked what they would change or improve about the *imi* application, participants expressed a desire for more content and content on additional topics:

I’d add more content, the content is slightly lacking for now.

I would also add a relationship section. Being in a relationship as a person in the LGBT community, there is a great need for knowledge on having thriving romantic, sexual, and even platonic relationships

They also expressed interest in the addition of social networking and other interactive features:

I’d like to be able to interact with more people, not just the automatic responses...

It would be cool if *imi* could track your location and find support groups in my area or a group chat to join with fellow lgbt people my age.

Another common critique was that the *imi* application felt tailored to youth beginning to explore their identities and less suitable for those who are already more affirmed:

I believe this resource is excellent for someone part of the LGBT community who is questioning themselves and actively needs help or would benefit from it. If you’re already out of the closet and comfortable with who you are, it may not be very beneficial.

### Preliminary Efficacy of the Intervention

#### Primary Outcome: Stress Appraisals

Stress appraisal improved over time across both arms. The treatment arm experienced significant improvements in challenge (t_121_=4.51; *P*<.001), threat (t_121_=–2.73; *P*=.007) and resource appraisals (t_121_=4.24; *P*<.001) from baseline to the end of the follow-up. The control arm also showed improvements in challenge (t_121_=1.96; *P*=.052) and resource appraisals (t_121_=2.83; *P*=.005; Table 2).

Controlling for baseline scores (Table 3), the treatment arm reported significantly higher challenge appraisals at follow-up than the control arm (Cohen *d*=0.25; coefficient for treatment arm *b*=0.26; *P*=.008). We did not observe a difference between the study arms for threat appraisals (*d*=0.10; *b*=–0.06; *P*=.37) or resource appraisals (*d*=0.15; *b*=0.14; *P*=.14).

**Table 2:** Within-arm changes in primary and secondary outcomes from baseline to the 4-week follow-up (N=244).

Outcomes			Control				Intervention			
			Baseline (n=135), mean (SD)	Follow-up (n=122), mean (SD)	*t* test (*df*)	*P* value^a^	Baseline (n=135), mean (SD)	Follow-up, (n=122), mean (SD)	*t* test (*df*)	*P* value^a^
**Primary outcomes**										
	**Stress appraisals**									
		Challenge	3.15 (0.88)	3.32 (0.93)	1.96 (121)	.052	3.30 (0.77)	3.64 (0.85)	4.51 (121)	<.001
		Threat	4.03 (0.72)	3.92 (0.69)	–1.79 (121)	.08	3.99 (0.73)	3.85 (0.77)	–2.73 (121)	.007
		Resource	3.42 (1.00)	3.67 (0.90)	2.83 (121)	.005	3.46 (0.98)	3.83 (0.92)	4.24 (121)	<.001
**Secondary outcomes**										
	**Cognitive and behavioral coping skills**									
		Self-distraction	3.20 (0.72)	3.23 (0.65)	0.25 (121)	.80	3.29 (0.70)	3.23 (0.65)	–1.33 (121)	.19
		Active coping	2.46 (0.82)	2.47 (0.72)	0.22 (121)	.83	2.46 (0.78)	2.65 (0.84)	1.80 (121)	.06
		Emotional support	2.31 (0.86)	2.43 (0.94)	1.88 (121)	.06	2.41 (0.89)	2.59 (0.94)	2.30 (121)	.02
		Instrumental support	2.27 (0.86)	2.30 (0.87)	0.65 (121)	.52	2.32 (0.95)	2.64 (0.90)	3.15 (121)	.002
		Venting	2.43 (0.83)	2.42 (0.82)	0.53 (121)	.57	2.43 (0.83)	2.48 (0.86)	0.91 (121)	.36
		Positive reframing	2.22 (0.88)	2.25 (0.78)	0.17 (121)	.87	2.23 (0.82)	2.45 (0.86)	2.98 (121)	.003
		Planning	2.57 (0.85)	2.56 (0.86)	–0.24 (121)	.81	2.59 (0.96)	2.79 (0.83)	2.80 (121)	.006
		Acceptance	2.79 (0.82)	2.73 (0.77)	–1.13 (121)	.26	2.78 (0.79)	2.87 (0.77)	0.70 (121)	.49
		Self-blame	3.04 (0.88)	2.76 (0.88)	–3.73 (121)	.003	3.07 (0.92)	2.91 (0.87)	–1.71 (121)	.09
		Substance use	1.42 (0.81)	1.38 (0.78)	–1.16 (121)	.25	1.27 (0.69)	1.33 (0.74)	0.63 (121)	.53
		Behavioral disengagement	2.16 (0.84)	1.99 (0.82)	–1.67 (121)	.10	2.13 (0.90)	2.01 (0.80)	–1.26 (121)	.21
	**Positive LGBTQ+^b^ identity**									
		Authenticity	5.06 (1.38)	5.08 (1.30)	0.20 (121)	.84	5.04 (1.38)	5.10 (1.33)	1.17 (121)	.24
		LGBTQ+ community	4.94 (1.35)	4.97 (1.18)	0.47 (121)	.64	4.69 (1.36)	4.82 (1.23)	1.44 (121)	.15
	**Internationalization of minority stress**									
		Internalization	3.21 (1.24)	2.94 (1.23)	–1.97 (121)	.051	3.29 (1.25)	3.19 (1.27)	–0.79 (121)	.43
	**Sense of belonging**									
		Thwarted belongingness	19.58 (6.12)	18.21 (6.24)	–2.53 (121)	.01	18.83 (6.29)	17.15 (6.99)	–3.53 (121)	<.001
	**Anxiety and depression symptoms**									
		Anxiety^c^	11.53 (5.31)	10.30 (5.78)	–2.55 (121)	.01	11.74 (5.44)	9.92 (5.56)	–4.42 (121)	<.001
		Depression^c^	12.40 (5.97)	11.45 (5.75)	–2.59 (121)	.01	13.00 (5.31)	11.61 (5.95)	–3.33 (121)	.001

^a^Paired *t* test.

^b^LGBTQ+: lesbian, gay, bisexual, transgender, queer, and other sexual and gender minority.

^c^Considered as continuous variables in the analyses.

**Table 3:** Between-arm differences in week 4 primary and secondary outcomes (N=244).

Outcomes			Cohen *d*	Modeling differences by arm^a^	
				Coefficients	*P* value
**Primary outcomes**					
	**Stress appraisals**				
		Challenge	0.25	0.26	.008
		Threat	0.1	–0.06	.37
		Resource	0.15	0.15	.14
**Secondary outcomes**					
	**Cognitive and behavioral coping skills**				
		Self-distraction	0.15	–0.04	.62
		Active coping	0.16	0.17	.07
		Emotional support	0.04	0.09	.37
		Instrumental support	0.24	0.29	.005
		Venting	0.03	0.05	.63
		Positive reframing	0.27	0.22	.02
		Planning	0.26	0.23	.02
		Acceptance	0.17	0.14	.13
		Self-blame	0.13	0.13	.18
		Substance use	0.16	0.05	.55
		Behavioral disengagement	0.02	0.02	.86
	**Positive LGBTQ+^b^ identity**				
		Authenticity	0.09	0.06	.57
		LGBTQ+ community	0.09	–0.002	.99
	**Internationalization of minority stress**				
		Internalization	0.07	0.15	.29
	**Sense of belonging**				
		Thwarted belongingness	0.08	–0.64	.34
	**Anxiety and depression symptoms**				
		Anxiety^c^	0.14	–0.55	.31
		Depression^c^	0.07	–0.17	.74

^a^The effect of study arm (*imi* vs *asterix*) on the outcome at follow-up controlling for the outcome at baseline.

^b^LGBTQ+: lesbian, gay, bisexual, transgender, queer, and other sexual and gender minority.

^c^Considered as continuous variables in the analyses.

#### Secondary Outcomes

##### Cognitive and Behavioral Coping Skills

At the 4-week follow-up, the treatment arm showed significant improvements in emotional support (t_121_=2.30; *P*=.02), instrumental support (t_121_=3.15; *P*=.002), positive reframing (t_121_=2.98; *P*=.003), and planning to cope (t_121_=2.80; *P*=.006). We observed no other changes over time in the other COPE subscales within the treatment arm. The control arm had significant reductions in self-blame (t_121_=–3.73; *P*=.003). We observed no other changes over time in the other COPE subscales within the control arm.

The effect of the intervention on cognitive and behavioral coping skills was greater among the treatment arm than the control arm (instrumental support: *d*=0.24, *b*=0.29, *P*=.005; positive reframing: *d*=0.27, *b*=0.22, *P*=.02; planning: *d*=0.26, *b*=0.23, *P*=.02). However, we observed no differences in emotional support (*d*=0.04; *b*=0.09; *P*=.37) or self-blame (*d*=0.13; *b*=0.13; *P*=.18) between the arms. We observed no other differences between the arms in the COPE subscales.

##### Positive LGBTQ+ Identity

We did not observe any significant changes over time in the authenticity or the community subscales of the LGB Positive Identity Measure in either arm, nor did we observe differences in improvements between the arms.

##### Internalization of Blame for Minority Stress

We did not observe reductions in internalization of blame for minority stress over time in either arm or differences between the two arms.

##### Sense of Belonging

At the 4-week follow-up, both the treatment arm (t_121_=–3.53; *P*<.001) and the control arm (t_121_=–2.53; *P*=.01) showed significant reductions in thwarted belongingness. We did not observe differences in reductions of thwarted belongingness between the arms (*d*=–0.08; *b*=–0.64; *P*=.34).

##### Anxiety and Depression Symptoms

We also observed reductions in anxiety and depression symptoms for both arms. Among the treatment arm, we found significant reductions in anxiety (t_121_=–4.42; *P*<.001) and depression (t_121_=–3.33; *P*=.001) from baseline to week 4. We found similar results among control arm participants (reductions in anxiety: t_121_=–2.55, *P*=.01; reductions in depression: t_121_=–2.35, *P*=.01). However, the treatment arm did not report significantly lower anxiety (*d*=0.14; *b*=–0.55; *P*=.31) or depression (*d*=0.07; *b*=–0.17; *P*=.74) at the follow-up than the control arm.

### Intervention Engagement

Approximately 98.5% (133/135) of the participants in the treatment arm and 97.8% (132/135) of the participants in the control arm created an account in their respective web resource within 4 weeks of being invited to access it. Participants in the treatment arm did not log significantly more sessions than participants in the control arm (t_268_=–1.84; *P*=.07); however, they spent significantly more time in the product (t_268_=–7.08; *P*<.001) and viewed more pages (t_268_=–10.30; *P*<.001). Similarly, although there were no significant differences between the treatment and control arms in thresholds of use for the number of sessions logged (≥5 sessions; *χ*^2^_1_=1.0; *P*=.39), significantly more participants in the treatment arm spent ≥10 minutes in the product (*χ*^2^_1_=49.2; *P*<.001) and viewed >10 unique pages (*χ*^2^_1_=101.9; *P*<.001) than participants in the control arm (Table 4). Although the treatment arm showed higher engagement than the control overall, the control arm participants clicked on more unique external links than the treatment arm (t_268_=4.51; *P*<.001) and were more likely to click on at least one external link (*χ*^2^_1_=13.1; *P*<.001).

**Table 4:** Engagement metrics by arm (N=270).

Engagement metrics		Control (n=135)	Intervention (n=135)	*P* value^a^
**Sessions**				
	Total sessions completed, median (range)	3 (0-19)	4 (0-18)	.07
	Low (0-4 sessions), n (%)	84 (62.2)	76 (56.3)	.39^b^
	High (≥5 sessions), n (%)	51 (37.8)	59 (43.7)	N/A^c^
**Time**				
	Total time spent (minutes), median (range)	3.08 (0.03-37.70)	12.14 (0.48-152.35)	<.001
	Low (0-10 minutes), n (%)	118 (87.4)	64 (47.4)	<.001^b^
	High (>10 minutes), n (%)	17 (12.6)	71 (52.6)	N/A
**Unique pages^d^**				
	Unique pages viewed, median (range)	5 (1-8)	13 (1-50)	<.001
	Low (0-10 pages), n (%)	135 (100)	61 (45.2)	<.001^b^
	High (>10 pages), n (%)	0 (0)	74 (54.8)	N/A
**External links**				
	Number of links clicked, median (range)	1 (0-14)	0 (0-6)	<.001
	None (0), n (%)	68 (50.4)	97 (71.9)	<.001^b^
	Any (>0), n (%)	67 (49.6)	38 (28.2)	N/A

^a^Student *t* test for continuous variables and chi-square tests for categorical variables.

^b^Compares dichotomized engagement (high vs low) and study arm (control vs intervention).

^c^N/A: not applicable. Refer to the *P* value for low engagement for a statistical comparison of high and low engagement by study arm.

^d^The maximum number of unique pages in the control web application (*asterix*) was 8 and the maximum for the *imi* application was 73.

### Engagement and Outcome Improvements Within the *imi* Application Arm

Exploratory analyses examining outcomes within the treatment arm revealed a positive relationship between the 3 engagement indicators and several of the primary and secondary outcomes (Table 5).

**Table 5:** Linear regressions examining differences in primary and secondary outcomes by engagement indicators among participants assigned to the *imi* application (n=122).

Outcomes				The number of sessions (high [≥5] vs low^a^)				Total minutes (high [>10] vs low^a^)				The number of unique pages viewed (high [>10] vs low^a^)		
				Coefficient		*P* value		Coefficient		*P* value		Coefficient		*P* value
**Primary outcomes**														
	**Stress appraisals**													
		Challenge	0.16		.25		0.42		.003		0.38		.008	
		Threat	0.03		.76		–0.13		.21		–0.13		.22	
		Resource	0.33		.03		0.50		.001		0.51		.001	
**Secondary outcomes**														
	**Cognitive and behavioral coping skills**													
		Self-distraction	0.17		.13		0.18		.11		0.07		.51	
		Active coping	0.15		.31		0.28		.06		0.18		.23	
		Emotional support	0.01		.95		0.32		.03		0.24		.10	
		Instrumental support	–0.03		.87		0.28		.06		0.17		.26	
		Venting	0.13		.36		0.24		.09		0.22		.13	
		Positive reframing	0.14		.34		0.08		.60		0.05		.76	
		Planning	–0.01		.94		0.25		.046		–0.01		.94	
		Acceptance	0.08		.52		0.10		.41		–0.01		.93	
		Self-blame	–0.02		.91		–0.32		.02		–0.22		.13	
		Substance use	–0.10		.41		–0.05		.67		–0.05		.66	
		Behavioral disengagement	–0.11		.42		–0.11		.39		–0.08		.54	
	Positive LGBTQ+^b^ identity													
		Authenticity	–0.03		.84		0.26		.12		–0.13		.44	
		LGBTQ+ community	0.11		.51		0.27		.09		0.03		.88	
	**Internationalization of minority stress**													
		Internalization	0.005		.98		–0.03		.90		0.20		.41	
	**Sense of belonging**													
		Thwarted belongingness	–1.51		.11		–1.30		.17		–0.21		.83	
	**Anxiety and depression symptoms**													
		Anxiety^c^	0.17		.82		–1.12		.13		–0.45		.55	
		Depression^c^	0.10		.90		–0.55		.48		0.23		.77	

^a^The effect of engagement (reached thresholds of use vs not reached) on the outcome at follow-up, controlling for outcome at baseline.

^b^LGBTQ+: lesbian, gay, bisexual, transgender, queer, and other sexual and gender minority.

^c^Considered as continuous variables in the analyses.

#### Sessions

Controlling for baseline scores, participants in the treatment group who engaged in ≥5 sessions during the intervention period had greater improvements in resource appraisals (*b*=0.33; *P*=.03) at follow-up than those who engaged in <5 sessions.

#### Total Minutes

Controlling for respective baseline scores, participants who spent >10 minutes in the *imi* application during the intervention period had significantly greater improvements in challenge appraisals (*b*=0.42, *P*=.003), resource appraisals (*b*=0.50; *P*=.001), and emotional support coping skills (*b*=0.32; *P*=.03) at follow-up than those who spent ≤10 minutes.

#### Unique Pages

Participants who viewed >10 unique pages within the *imi* application during the intervention period had significantly greater improvements in challenge appraisals (*b*=0.38, *P*=.008) and resource appraisals (*b*=0.51; *P*=.001) at follow-up than those who viewed ≤10 pages, controlling for baseline scores.

## Discussion

### Principal Findings

Digital interventions show promise in supporting the mental health of SGM youth, given the appeal of the modality, their suitability to deliver engaging asynchronous content, and their unique capacity to reach large numbers of SGM youth, including youth who may be unable to access in-person services because of transportation concerns, availability of local services, or concerns about privacy and stigma. In this study, we found high acceptability for both the full *imi* intervention web application and the resource page–only control, with participants reporting greater satisfaction and engagement with the *imi* web application. SGM youth assigned to the *imi* web application also had greater improvements in stress appraisals and coping skills. Given the potential for digital interventions such as the *imi* application to support the mental health needs of SGM youth, we discuss our findings in detail in the following sections and note opportunities to advance work in this area.

Participants indicated that the *imi* application’s content was acceptable and well-suited for SGM youth populations. Consistent with our hypothesis, participants randomly assigned to receive the *imi* application were more likely than those assigned to the resource-only control arm to report a positive experience with the intervention and to recommend it to SGM youth friends. These sentiments were echoed in the qualitative feedback, in which participants highlighted the benefits of viewing the stories of other SGM youth and learning approaches for coping with stress. Participants also offered several ways of improving the *imi* application, including developing greater breadth (eg, more overall content) and depth (eg, expansion to include a section focused on LGBTQ+ relationships) of content, and the addition of new features (eg, the ability to interact with other SGM youth through the web application). Collectively, these findings align with the growing body of evidence suggesting that SGM youth consider digital interventions to be acceptable modalities through which to receive mental health support [18,19,24]. They also point to concrete directions for intervention improvement.

For individuals to respond effectively against a stressor, they must be able to feel confident in their ability to address it [43]. Consistent with our hypothesis, SGM youth in the *imi* application arm were more likely than their peers in the control arm to appraise stressful situations as a surmountable challenge at the 4-week follow-up. The *imi* application arm was also less likely to report appraising stressful events as having lasting, negative repercussions. Participants in both study arms reported gains in having the internal and external resources to deal with stressful situations. The absence of a significant difference in resource appraisals between the 2 arms may be explained by the fact that both sites contained the same *imi-*based curated list of resources tailored to SGM youth. Taken together, these findings are promising and suggest that the *imi* application may help SGM youth situate stressors in their lives as transient and addressable, which, in turn, may reduce the acuity of these experiences on their mental health.

We also observed important initial changes in SGM youth’s coping skills after the 4-week follow-up period. Compared with the control arm, participants in the *imi* application arm reported greater gains in their ability to seek out instrumental support, positively reframe challenging situations, and engage in planning as coping skills. Interestingly, we observed improvements in measured areas of mental health across both arms (eg, anxiety and depression symptoms), suggesting that web applications of varying scope tailored to SGM youth are promising sources of mental health support. Unfortunately, the absence of a no-treatment control group prevents us from inferring whether both interventions are efficacious relative to the absence of any program. Although comparing the full *imi* intervention with a resource-only subset of the *imi* website (ie, *asterix*) makes it harder to detect differences between the arms, we felt that withholding referrals to care for SGM youth would be unethical. We also selected this control as we wished to examine whether the full, interactive *imi* intervention provides benefits to SGM youth that go above and beyond what might be gained by being given access to a unified, vetted list of freely available existing resources. Previous studies suggest that SGM youth are increasingly and disproportionately searching for support on the web; however, this opens them to increased experiences of encountering homophobic and transphobic content and other hate speech [23]. It may be the case that gathering vetted resources in a single safe site designed specifically for them (ie, the resources section of the *imi* application) supports the well-being of SGM youth in and of itself. Although our design lacked a no-treatment control and, therefore, was unable to test this hypothesis, it does highlight the additive benefit of interactive identity affirmation and coping content contained in the full *imi* intervention; that is, although participants who were assigned both versions of the web application (the resource-only version of *imi* called *asterix* and the full interactive *imi* intervention) experienced decreased feelings of anxiety and depression after 4 weeks, only those given the full intervention experienced the additional benefit of greater stress appraisal and cognitive and behavioral coping skill improvements.

### Future Directions

The lack of observed differences between the arms across other domains (eg, identity affirmation and connectedness to the LGBTQ+ community, internalization of minority stress, and sense of belonging) may be related to several additional considerations. First, it is possible that the 4-week study period may be too short to allow for meaningful changes to emerge in certain types of outcomes. For example, it may be that identity-related shifts require more time to manifest. Second, consistent with participants’ qualitative feedback, it is possible that SGM youth require a greater amount of content and activities in a web application to, for example, feel more connected to the broader LGBTQ+ community. Third, some participants noted in their qualitative recommendations that the *imi* application may be most useful for SGM youth who are earlier in the exploration and affirmation of their identities. However, it remains unclear whether the intervention’s effects may be greater for SGM youth earlier in their coming out process or those questioning their identities, a question which this study was not statistically powered to address. Future research is needed to examine whether the intervention’s effect on the outcomes requires greater content to be developed within the web application, whether the intervention effects are moderated by how comfortable users are with their identities, or whether a longer follow-up period may allow for changes in distal outcomes to be observed.

Participants’ engagement with the web application also offers insights into its acceptability and preliminary efficacy. Most participants across both arms created an account on their assigned web application, with participants reporting a comparable number of log-ins over the 4-week follow-up period. As expected, participants in the *imi* application arm viewed more unique pages than participants in the control arm. In and of itself, this finding does not indicate greater engagement with the *imi* application, as the control web application contained only a small subset of pages from the *imi* application. Consistent with our hypothesis, participants in the *imi* application arm also spent more time logged into the intervention than their peers in the control arm. However, *imi* participants were less likely than their control counterparts to click on external links. When taken together, these findings may be explained by two alternate (but complementary) possibilities: *imi* participants may have been more likely to spend time on the web application and less likely to require clicking on external links as the created content and activities were engaging and sufficiently helpful. Alternatively, given the limited content available to SGM youth in the control arm, participants assigned to *asterix* may have spent their time reviewing the content offered through the links to curated LGBTQ+-affirming resources, a possibility we could not assess in this study as we could not track participants’ behavior on external websites. Future research examining participants’ experiences within and outside of the web application may be warranted.

As an exploratory analysis, we examined whether SGM youth’s engagement with the *imi* application could affect the strength of the intervention’s effect. Consistent with our hypothesis, and in alignment with prior research examining the role of engagement on a digital intervention’s efficacy [41,44,45], higher engagement with the *imi* application (eg, ≥10 minutes spent on the tool; >10 unique pages viewed) was linked to greater improvement on our primary outcome variables. This finding supports the interpretation that engaging with the *imi* application’s content improves coping abilities. On the basis of our engagement data, it is promising to see that more than half of the participants assigned to the full *imi* intervention achieved these thresholds. However, it is worthwhile noting that SGM youth were incentivized for creating an account within the tool and sent reminders by the study team to log back in over time. It remains crucial to understand whether these thresholds and their associated benefits persist outside of the clinical trial setting. Efforts to explore engagement in a naturalistic study may be worthwhile to examine and affirm external validity.

### Strengths and Limitations

Several limitations are worth noting. First, although the intervention effects moved in favor of the *imi* application arm in primary and secondary outcomes, our ability to detect these effects with statistical precision was limited by our small sample size and short follow-up period. Future scaled-up versions of the *imi* intervention with larger sample sizes, a longer duration, and a greater number of follow-up periods may be warranted to examine efficacy and effectiveness with greater precision. Second, some of the indicators used to measure our outcomes (eg, authenticity and LGBTQ+ community connectedness) were originally developed with adult populations. Given the unique needs of SGM youth, it is possible that the measures used in our study were not optimal for use with SGM youth populations. Future research examining the psychometric appropriateness of these measures with SGM youth populations may be warranted. Third, our ability to recruit youth from the lowest end of the age spectrum was somewhat limited. Only 11.1% (30/270) of the sample was aged 13 to 14 years. This is a common limitation in studies of SGM youth [46]. However, given the qualitative feedback that youth earlier in the journey of identity exploration may benefit more from the tool, future implementation research should explore pathways for making the tool more accessible to both younger youth and youth who may be in the earlier stages of identity exploration or questioning their identities. Similarly, although the diversity of race and ethnicities, as well as sexual and gender identities represented in our sample, is a strength, this initial pilot study was not powered to assess whether the intervention has differential efficacy based on these demographic characteristics. Finally, we weighed the advantages and disadvantages of enforcing a type I error (ie, false positive) correction relative to a type II error (ie, false negative). Given the exploratory nature of our trial and the small sample size of this pilot study, we did not include a family-wise error rate correction, which is justified in early exploration [47]. Future research examining the effects of both the *imi* and *asterix* applications with larger and population-representative samples may be warranted.

Our study also has several strengths worth emphasizing. First, it is one of the few clinical trials examining how to design and deliver a coping intervention that may reduce the negative effects of minority stress on SGM youth. Second, compared with face-to-face programs for SGM youth and synchronous digital interventions, our findings underscore the feasibility and acceptability of an asynchronous digital intervention that overcomes access and engagement barriers by being freely accessible on demand, scalable, confidential, and not requiring a significant time commitment. Our study demonstrated that a brief (as little as 10 minutes), self-guided intervention may have significant benefits for coping with stress. Finally, compared with prior trials, our study increases the likelihood that the findings are generalizable, given our commitment to recruiting and retaining a diverse group of SGM youth across races, ethnicities, sexual orientations and gender identities, geographies, and socioeconomic backgrounds.

### Conclusions

This study demonstrated that a brief web-based intervention can provide self-guided, asynchronous, and confidential support that improves the ability of SGM youth to cope with minority stress. As a tool, the *imi* application may provide public health utility and value by expanding the reach and scalability of mental health programs, particularly for SGM youth who may be unable to participate in time-intensive, synchronous interactions. It may also serve as an ancillary tool for community-based agencies seeking to engage their SGM youth clients via the internet. Further research is needed to examine the long-term effects of the *imi* application and its potential for scalability and population health impact.

## Appendix

Multimedia Appendix 1 Informed consent form.

Multimedia Appendix 2 The *imi* application informational video.

Multimedia Appendix 3 Screenshots from the *imi* (treatment) and *asterix* (control) web applications.

Multimedia Appendix 4 Assessment of attrition and differential attrition by study arm.

Multimedia Appendix 5 CONSORT eHEALTH Checklist (V 1.6.1).

## Abbreviations

COPE: Coping Orientation to Problems Experienced
LGB: lesbian, gay, and bisexual
LGBTQ+: lesbian, gay, bisexual, transgender, queer, and other sexual and gender minority
NPS: net promoter score
SGM: sexual and gender minority
