# Supplementary material for: An Identity-Affirming Web Application to Help Sexual and Gender Minority Youth Cope With Minority Stress: Pilot Randomized Controlled Trial
Source: J Med Internet Res. 2022 Aug 1;24(8):e39094. doi: 10.2196/39094 (PMC9379807; doi:10.2196/39094)
Supplement: Multimedia Appendix 1 [file jmir_v24i8e39094_app1.docx]

**University of Pennsylvania**

**Research Participant Informed Consent Form**

Version 10/14/2021

| **Protocol Title:** | Piloting a Web App for Sexual and Gender Minority Youth Mental Health |
| --- | --- |
| **Principal Investigator:** | Dr. José Bauermeister  Department of Family and Community Health  University of Pennsylvania School of Nursing  418 Curie Blvd, Room 222L  Philadelphia, PA 19104  215-898-9993  bjose@upenn.edu |
| **Study Contact:** | Jesse Golinkoff  215-746-5529  agolinko@upenn.edu |
| **Sponsor** | Hopelab |

## **Research study summary**

You are being invited to participate in a research study. Your participation is voluntary, and you should only participate if you completely understand what the study requires and what the risks of participation are.

You should ask the study team any questions you have related to participating before agreeing to join the study. If you have any questions about your rights as a human research participant at any time before, during or after participation, please contact the Institutional Review Board (IRB) at (215) 898-2614 for assistance.

The research study is being conducted to evaluate digital tools designed to provide mental health and wellbeing resources relevant to LGBTQ+ youth in the United States. If you agree to join the study, you will be asked to complete two surveys (initial survey and follow-up survey on day 28) and to use a web-based digital resource. You can access the web resource on any computer, smartphone, or tablet that has internet access.

Your participation will last for 28 days.

You may not benefit personally from being in this research study. There are very few risks involved in this study. Some questions that you will be asked in the surveys might make you feel uncomfortable. You are free to decline or stop participation at any time during or after the initial consenting process.

# **Why am I being asked to participate?**

You are being asked to take part in a research study. Your participation is voluntary which means you can choose whether or not to participate. If you decide to participate or not to participate there will be no loss of benefits to which you are otherwise entitled.

Before you make a decision, you will need to know the purpose of the study, the possible risks and benefits of being in the study, and what you will have to do if you decide to participate.

# **What is the purpose of the study?**

The purpose of this study is to evaluate digital tools designed to provide mental health and wellbeing information and resources relevant to LGBTQ+ youth in the United States. The purpose of The Digital Wellbeing Project is to provide information and resources about LGBTQ+ identities, mental health challenges, and stigma.

If you agree to be in this study, you will be randomly assigned to one of two study groups. The groups will receive access to two different web-based digital resources, one of which has more content available than the other.

Your use of the web resource and the feedback you provide will help us to improve the resource and make it more inclusive to the diverse needs of the entire community. You are being asked to be in this study because, as a young LGBTQ+ person, you can help us determine whether the information and tools on the web resource address your needs and help you cope with life stressors.

Your feedback will help us assess the web resource to make sure it’s easy to use, that the information makes sense, and, most importantly, to make sure the resource will be relevant to users.

**How long will I be in the study?**

The study will take place over a period of 28 days.

# **How many other people will be in the study?**

If you decide to participate, you will be one of approximately 300 people in this research study.

# **What will I be asked to do?**

If you agree to be in this study, you will be randomly assigned to one of two study groups. One of the two groups will have more interactive web-based digital resource content available to them.

You will be asked to complete a 30- to 40-minute initial survey. Some of the questions in the survey are sensitive in nature. You are asked to respond to all questions honestly. All the information you provide will remain confidential.

After the initial survey, you will be sent a link to the web-based digital resource to register for your account. You can access the resource on any computer, smartphone, or tablet that has internet access. All participants are encouraged to log into the digital resource at least twice a week.

We will be able to track where you go within the resource, what features you use, what content you see, and the length of time you spend in the resource. You will be asked to complete a 30- to 40- minute follow-up survey 28 days after your initial survey. The survey time points are below:

- Initial survey (today)
- 28 days after you finish the initial survey

Depending on the communication preferences you indicate on the initial survey, you may receive study-related emails, phone calls, text messages, or direct messages via social media asking you to complete surveys.

Any communication you receive from us will not include information that you have shared with us in surveys or in the web resource, nor will they contain other sensitive or confidential information. All communications from the Digital Wellbeing Project study team will be sent over a secure, encrypted, and Health Insurance Portability and Accountability Act-compliant (HIPAA) server.

# **Will I be paid for being in this study?**

You may receive up to $70 in digital gift cards for participating in all aspects of this study. Digital gift cards (i.e. Amazon e-gift cards) will be sent to your email address.

- Initial survey and account creation on the web-based digital resource: $30
- Day 28 survey: $40

# **What are the risks?**

Many measures will be taken to ensure your confidentiality and privacy during this study. However, we want to make you aware of some potential risks or discomforts. We also ask that you take active steps to protect your privacy.

*Potential risks of data collection and loss of confidentiality*

Survey responses will be stored on a secure server with strict access controls. To protect your confidentiality, no identifying information about you will appear in the data files used in the study. We will make every effort to protect your confidentiality, but there is a small possibility that information you provide to us could become known to others.

*Potential risks of using a web-based digital resource for a research study*

You may be uncomfortable logging onto the web-based digital resource or having others see you log onto it. We suggest that you log in from a place you feel safe and comfortable. You must not share your login information with others.

If someone other than you sees the web resource or the survey questions you’re asked to complete, they may know that you are taking part in a research study about LGBTQ+ mental health and wellbeing. This would result in a breach of confidentiality for you. We encourage you to protect your devices with a password or PIN and to delete messages sent from the study team after you have read them to minimize the risk of someone finding out about your participation in this study.

*Potential risks of study communication and loss of confidentiality*

Study-related messages will be sent to you from study staff via email. Depending on the communication methods you specify in the initial survey, you may also receive study-related communication by text message and/or direct message via social media. The messages will mainly be reminders for you to log onto the web resource or to take your follow-up survey.

To make sure no one with access to your phone can read your text messages and breach your confidentiality, you should protect your phone with a password or PIN and change your text message settings so the content of the messages does not appear on the main screen when you receive a message.

To further protect your privacy, we recommend you delete the text messages and direct messages via social media after you have read them. If you have not completed a survey after 7 days, we may call you to remind you. We will ask questions to confirm your identity before discussing anything related to the study. If you do not pick up the call, we will not leave a voicemail.

**How will I benefit from the study?**

You may not benefit personally from being in this research study. However, we believe that the content and information provided in the web resource will help LGBTQ+ youth across the United States better address their mental health and wellbeing needs.

Depending on the study group to which you are assigned, you may receive more resources and have access to activities. We want to see which version of the web-based digital resource is most successful at communicating necessary mental health and wellbeing information.

Regardless of the group to which you are assigned, you will receive relevant mental health information and resources to help you better take care of yourself.

# **What happens if I do not choose to join the research study?**

You may choose to join the study or you may choose not to join the study. Your participation is voluntary. There is no penalty if you choose not to join the research study. You will lose no benefits or advantages that are now coming to you, or would come to you in the future.

# **When is the study over? Can I leave the study before it ends?**

The study is expected to end after all participants have completed all study activities. The study may be stopped without your consent for the following reasons:

- The Principal Investigator feels it is best for your safety and/or health (you will be informed of the reasons why)
- You have not followed the study instructions
- The Principal Investigator or the Office of Regulatory Affairs at the University of Pennsylvania can stop the study at any time

You have the right to drop out of the research study at any time during your participation, without penalty. If you no longer wish to be in the research study, please send Jesse Golinkoff an email at agolinko@upenn.edu and write that you would like to withdraw from the study.

**How will my personal information be protected during the study?**

Every effort will be taken to protect your identity as a participant in this study. The information you share in the surveys you take is confidential and will not be linked to your name in any report or publication of this study or its results. Instead, you will be known only through a Study ID number.

Any data linking your first name, email address, or phone number to your Study ID number will be kept on a secure server. Any personally identifiable data that are collected from you will be stored on a secure, confidential, and HIPAA-compliant server housed at the University of Pennsylvania. De-identified data from the study will be kept indefinitely for secondary analyses.

Any study-related messages that are sent to you will be free of any identifying information and will not have any of your personal health information. To further ensure your privacy, you can change the settings on your phone to keep the body of text messages from appearing on the main screen when you receive a message.

Please be aware that if you choose to receive text message communication but are not the account holder on your phone (i.e, you’re on a family plan), then whoever is the account holder may be able to see that you were contacted via text message; however, they will not have access to the text message.

Standard messaging rates for your phone carrier will apply. All messages generated by the study are sent and stored on a HIPAA-compliant server. Messages can only be directly linked to your Study ID and first name.

The research team has experience safeguarding data. All staff will be trained and required to sign certifications of confidentiality before working with any sensitive information. Staff training is extensive and is not only about confidentiality, but also about respect for you and your safety. Additionally, all data will be secured using an SSL 256-bit encryption. SSL encryption is the standard for all web-based transactions.

You should understand that in all cases we will take the necessary action, including reporting to authorities, to prevent serious harm to yourself or others. For example, in the case of someone mentioning they will attempt suicide or threatening the life of another person.

**Who will have access to my data?**

The information you share in the study surveys will be accessible only to the research team. The study's technology partner, Hopelab, will have access to your first name, email address, and phone number, but will not have access to your survey data.

# **What may happen to my information collected in this study?**

We plan to publish the results of this study but will not include any information that could identify you. Your email address, phone number, and any other personal identifiers will be stored on a password-protected encrypted server. This information will be destroyed five years after the study is completed. Your survey answers will not be tied to this personal information.

All your survey answers will be kept on password-protected computers and servers. By consenting to this research, you allow the researchers to keep and analyze de-identified study data indefinitely (de-identified data has no chance of being re-identified).

## **Future use of data**

Your information will be de-identified. De-identified means that all identifiers have been removed. The information could be stored and shared for future research in this de-identified fashion. The information may be shared with other researchers within Penn, or other research institutions.

It would not be possible for future researchers to identify you as we would not share any identifiable information about you with future researchers. Sharing de-identified data can be done in the future without again seeking your consent, as permitted by law. The future use of your information only applies to the information collected on this study.

#

# **Who can I call with questions, complaints or if I’m concerned about my rights as a research subject?**

If you have questions, concerns or complaints regarding your participation in this research study or if you have any questions about your rights as a research subject, you should speak with the Principal Investigator listed on page one of this form.

If a member of the research team cannot be reached or you want to talk to someone other than those working on the study, you may contact the Office of Regulatory Affairs at the University of Pennsylvania with any question, concern, or complaint by calling (215) 898-2614.

**Consent**

Please indicate below if you agree to be in this study. By agreeing to participate in this study, you will not give up any of your legal rights. A copy of the consent form will be available to you in the web-based digital resource.

Do you agree to participate in the study?

- Yes, I agree to participate in the study
- No, I do not agree to participate in the study

Do you agree to allow us to keep your email address and contact you about future research opportunities in the next five years?

- Yes, you may keep my email address for future research opportunities
- No, you may not keep my email address for future research opportunities
