# Supplementary figures and images for: An Identity-Affirming Web Application to Help Sexual and Gender Minority Youth Cope With Minority Stress: Pilot Randomized Controlled Trial
Source: J Med Internet Res. 2022 Aug 1;24(8):e39094. doi: 10.2196/39094 (PMC9379807; doi:10.2196/39094)

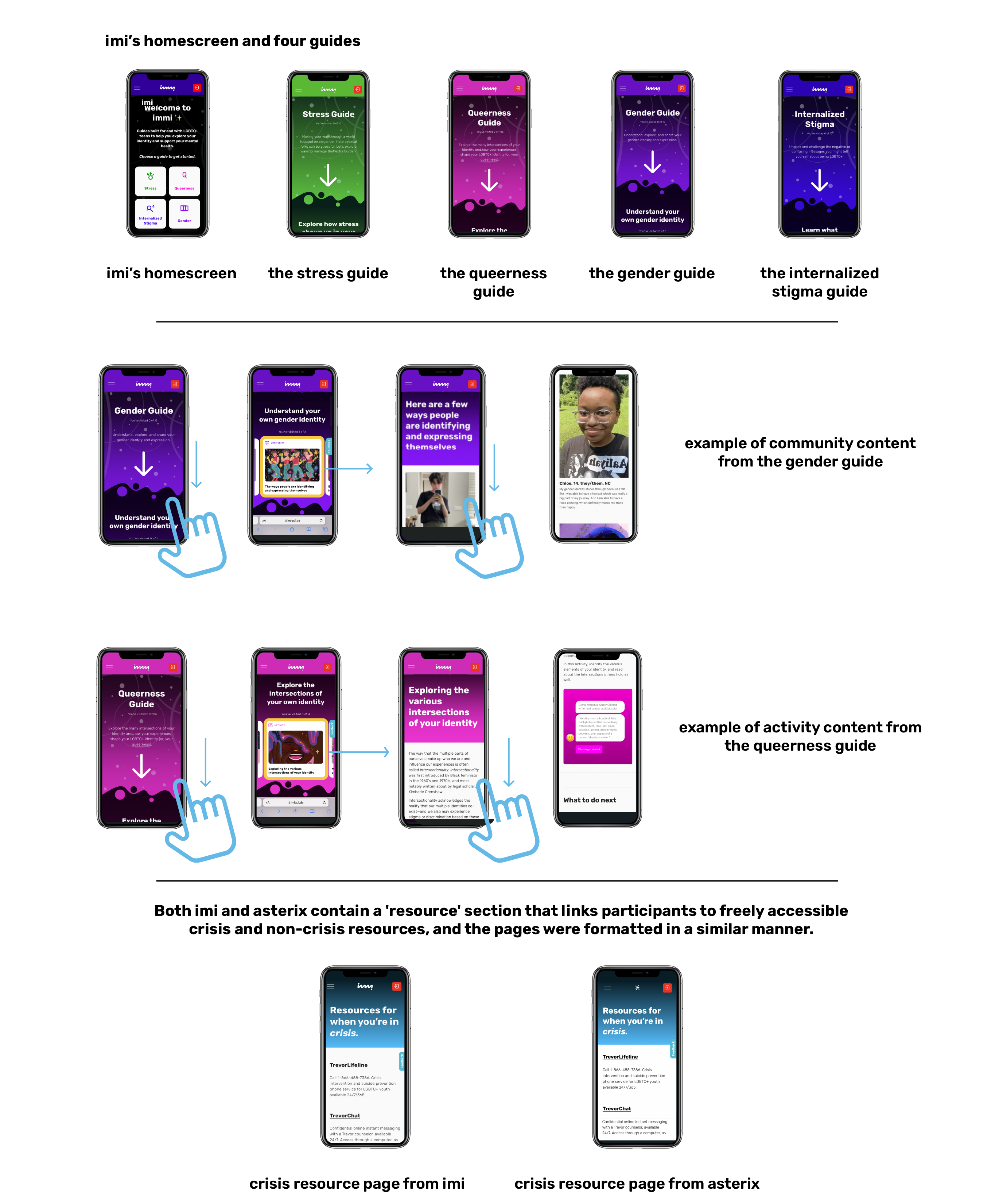

Supplement: Multimedia Appendix 3 [file jmir_v24i8e39094_app3.png]
