# Supplementary material for: An Identity-Affirming Web Application to Help Sexual and Gender Minority Youth Cope With Minority Stress: Pilot Randomized Controlled Trial
Source: J Med Internet Res. 2022 Aug 1;24(8):e39094. doi: 10.2196/39094 (PMC9379807; doi:10.2196/39094)
Supplement: Multimedia Appendix 4 [file jmir_v24i8e39094_app4.docx]

Multimedia Appendix 3. Assessment of attrition and differential attrition by study arm.

|  | Attrition | | Differential Attrition | | | | |
| --- | --- | --- | --- | --- | --- | --- | --- |
|  | All (N=270) | | Control (n=135) | | Intervention (n=135) | |  |
|  | Did not complete follow-up  (n=26) | Complete follow-up (n=244) | Did not complete follow-up (n=13) | Complete follow-up (n=122) | Did not complete follow-up (n=13) | Complete follow-up (n=122) |  |
| ***Demographics Characteristics*** |  |  |  |  |  |  |  |
| **Age (years), mean (SD)** | 15.42 (1.53) | 16.60 (1.44) | 15.46 (1.66) | 16.52 (1.47) | 15.38 (1.45) | 16.68 (1.41) |  |
| **Geographic region, n (%)** | | | | | | |  |
| Metropolitan | 24 (92.3%) | 225 (92.2%) | 12 (92.3%) | 114 (93.4%) | 12 (92.3%) | 111 (91.0%) |  |
| Micropolitan | 1 (3.9%) | 13 (5.3%) | 0 (0%) | 7 (5.7%) | 1 (7.7%) | 6 (4.9%) |  |
| Small town | 1 (3.9%) | 3 (1.2%) | 1 (7.7%) | 0 (0%) | 0 (0%) | 3 (2.5%) |  |
| Rural areas | 0 (0%) | 3 (1.2%) | 0 (0%) | 1 (0.8%) | 0 (0%) | 2 (1.6%) |  |
| **Census region, n (%)** | | | | | | |  |
| Northeast | 2 (7.7%) | 35 (14.3%) | 1 (7.7%) | 15 (12.3%) | 1 (7.7%) | 20 (16.4%) |  |
| Midwest | 4 (15.4%) | 49 (20.1%) | 2 (15.4%) | 27 (22.1%) | 2 (15.4%) | 22 (18.0%) |  |
| South | 10 (38.5%) | 83 (34.0%) | 5 (38.5%) | 41 (33.6%) | 5 (38.5%) | 42 (34.4%) |  |
| West | 10 (38.5%) | 77 (31.6%) | 5 (38.5%) | 39 (32.0%) | 5 (38.5%) | 38 (31.2%) |  |
| **Education, n (%)** | | | | | | |  |
| K-5th grade | 1 (3.9%) | 2 (0.8%) | 1 (7.7%) | 1 (0.8%) | 0 (0%) | 1 (0.8%) |  |
| 6-8th grade | 11 (42.3%) | 87 (35.7%) | 4 (30.8%) | 44 (36.1%) | 7 (53.9%) | 43 (35.2%) |  |
| 9-11th grade | 11 (42.3%) | 89 (36.5%) | 7 (53.9%) | 44 (36.1%) | 4 (30.8%) | 45 (36.9%) |  |
| High school diploma or equivalent | 2 (7.7%) | 50 (20.5%) | 1 (7.7%) | 25 (20.5%) | 1 (7.7%) | 25 (20.5%) |  |
| Some post-secondary education | 1 (3.9%) | 16 (6.6%) | 0 (0%) | 8 (6.5%) | 1 (7.7%) | 8 (6.6%) |  |
| **Subjective SES, n (%)** |  |  |  |  |  |  |  |
| Wealthy | 0 (0%) | 1 (0.4%) | 0 (0%) | 1 (0.8%) | 0 (0%) | 0 (0%) |  |
| Upper-middle class | 8 (30.8%) | 35 (14.3%) | 3 (23.1%) | 15 (12.3%) | 5 (38.5%) | 20 (16.4%) |  |
| Middle class | 11 (42.3%) | 108 (42.3%) | 6 (46.2%) | 51 (41.8%) | 5 (38.5%) | 57 (46.7%) |  |
| Working class | 3 (11.5%) | 58 (23.8%) | 1 (7.7%) | 32 (26.2%) | 2 (15.4%) | 26 (21.3%) |  |
| Low income or poor | 3 (11.5%) | 28 (11.5%) | 3 (23.1%) | 16 (13.1%) | 0 (0%) | 12 (9.8%) |  |
| I prefer not to respond | 1 (3.9%) | 14 (5.7%) | 0 (0%) | 7 (5.7%) | 1 (7.7%) | 7 (5.7%) |  |
| **Sex at birth, n (%)** | | | | | | |  |
| Male | 3 (11.5%) | 58 (23.8%) | 2 (15.4%) | 30 (24.6%) | 1 (7.7%) | 28 (22.9%) |  |
| Female | 33 (88.5%) | 186 (76.2%) | 11 (84.6%) | 92 (75.4%) | 12 (92.3%) | 94 (77.1%) |  |
| **Living status, n (%)** | | | | | | |  |
| Living with parent(s)/guardian(s) | 2 (7.7%) | 46 (18.8%) | 1 (7.7%) | 25 (20.5%) | 1 (7.7%) | 21 (17.2%) |  |
| Other | 24 (92.3%) | 198 (81.2%) | 12 (92.3%) | 97 (79.5%) | 12 (92.3%) | 101 (82.8%) |  |
| **Race, n (%)** | | | | | | |  |
| Racial/ethnic minority | 22 (84.6%) | 188 (77.0%) | 12 (92.3%) | 95 (77.9%) | 10 (76.9%) | 93 (76.2%) |  |
| Exclusively ID as Non-Hispanic White | 4 (15.4%) | 56 (23.0%) | 1 (7.7%) | 27 (22.1%) | 3 (23.1%) | 29 (23.8%) |  |
| **Multiple races, n (%)** | | | | | | |  |
| Multiple races/ethnicities | 12 (46.1%) | 71 (29.1%) | 6 (46.1%) | 38 (31.1%) | 6 (46.1%) | 33 (27.0%) |  |
| Single race/ethnicity | 14 (53.9%) | 173 (70.9%) | 7 (53.9%) | 84 (68.9%) | 7 (53.9%) | 89 (73.0%) |  |
| **Gender identity** ^a^**, n (%)** | | | | | | |  |
| Transgender | 5 (19.2%) | 53 (21.7%) | 3 (23.1%) | 23 (18.8%) | 2 (15.4%) | 30 (24.6%) |  |
| Non-binary | 8 (30.8%) | 74 (30.3%) | 5 (38.5%) | 37 (30.3%) | 3 (23.1%) | 37 (30.3%) |  |
| Cisgender | 10 (38.5%) | 97 (39.8%) | 4 (30.8%) | 51 (41.8%) | 6 (46.1%) | 46 (37.7%) |  |
| Other | 3 (11.5%) | 20 (8.2%) | 1 (7.7%) | 11 (9.0%) | 2 (15.4%) | 9 (7.4%) |  |
| **Gender identity, n (%)** |  |  |  |  |  |  |  |
| Not questioning | 19 (73.1%) | 199 (81.6%) | 8 (61.5%) | 99 (81.2%) | 11 (84.6%) | 100 (82.0%) |  |
| Questioning | 7 (26.9%) | 45 (18.4%) | 5 (38.5%) | 23 (18.9%) | 2 (15.4%) | 22 (18.0%) |  |
| **Multiple gender identities, n (%)** | | | | | | |  |
| Multiple identities | 11 (42.3%) | 102 (41.8%) | 7 (53.9%) | 49 (40.2%) | 4 (30.8%) | 53 (43.4%) |  |
| Single identity | 15 (57.7%) | 142 (58.2%) | 6 (46.1%) | 73 (59.8%) | 9 (69.2%) | 69 (56.6%) |  |
| **Sexual orientation** ^b^**, n (%)** |  |  |  |  |  |  |  |
| Plurisexual | 10 (38.5%) | 115 (47.1%) | 8 (61.5%) | 55 (45.1%) | 2 (15.4%) | 60 (49.2%) |  |
| Gay or Lesbian | 4 (15.3%) | 48 (19.7%) | 2 (15.4%) | 27 (22.1%) | 2 (15.4%) | 21 (17.2%) |  |
| Other | 12 (46.2%) | 81 (33.2%) | 3 (23.1%) | 40 (32.8%) | 9 (69.2%) | 41 (33.6%) |  |
| **Sexual orientation, n (%)** | | | | | | |  |
| Not questioning | 18 (69.2%) | 207 (84.8%) | 9 (69.2%) | 101 (82.8%) | 9 (69.2%) | 106 (86.9%) |  |
| Questioning | 8 (30.8%) | 37 (15.2%) | 4 (30.8%) | 21 (17.2%) | 4 (30.8%) | 16 (13.1%) |  |
| **Multiple sexual orientations, n (%)** | | | | | | |  |
| Multiple identities | 11 (42.3%) | 96 (39.3%) | 6 (46.1%) | 48 (39.3%) | 5 (38.5%) | 48 (39.3%) |  |
| Single identity | 15 (57.7%) | 148 (60.7%) | 7 (53.9%) | 74 (60.7%) | 8 (61.5%) | 74 (60.7%) |  |
| **Pronouns** ^c^**, n (%)** | | | | | | |  |
| They/They+ | 16 (61.5%) | 112 (45.9%) | 9 (69.2%) | 55 (45.1%) | 7 (53.9%) | 57 (46.7%) |  |
| He/He+ or She/She+ | 10 (38.5%) | 117 (47.9%) | 4 (30.8%) | 59 (48.4%) | 6 (46.1%) | 58 (47.5%) |  |
| Other | 0 (0%) | 15 (6.2%) | 0 (0%) | 8 (6.5%) | 0 (0%) | 7 (5.7%) |  |
| **Multiple pronouns, n (%)** | | | | | | |  |
| Multiple sets of pronouns | 13 (50.0%) | 87 (35.7%) | 6 (46.1%) | 39 (42.0%) | 7 (53.9%) | 48 (39.3%) |  |
| Single set of pronouns | 13 (50.0%) | 157 (64.3%) | 7 (53.9%) | 83 (68.0%) | 6 (46.1%) | 74 (60.7%) |  |
| **Outness, n (%)** | | | | | | |  |
| Definitely in the closet | 2 (7.7%) | 22 (9.0%) | 1 (7.7%) | 11 (9.0%) | 1 (7.7%) | 11 (9.0%) |  |
| In the closet most of the time | 5 (19.2%) | 42 (17.2%) | 2 (15.4%) | 17 (13.9%) | 3 (23.1%) | 25 (20.5%) |  |
| Half in the closet, half out of the closet | 10 (38.5%) | 83 (34.0%) | 6 (46.2%) | 45 (36.9%) | 4 (30.8%) | 38 (31.2%) |  |
| Out of the closet most of the time | 6 (23.1%) | 70 (28.7%) | 2 (15.4%) | 31 (25.4%) | 4 (30.8%) | 39 (32.0%) |  |
| Completely out of the closet | 3 (11.5%) | 27 (11.1%) | 2 (15.4%) | 18 (14.8%) | 1 (7.7%) | 9 (7.4%) |  |
| ***Primary Outcomes*** | | | | | | |  |
| **Stress appraisals, mean (SD)** | | | | | | |  |
| Challenge | 3.14 (0.84) | 3.23 (0.83) | 2.90 (0.84) | 3.17 (0.88) | 3.38 (0.80) | 3.29 (0.77) |  |
| Threat | 4.05 (0.99) | 4.00 (0.69) | 4.19 (1.03) | 4.01 (0.68) | 3.92 (0.96) | 4.00 (0.70) |  |
| Resource | 3.41 (1.17) | 3.44 (0.97) | 3.26 (1.04) | 3.43 (1.00) | 3.56 (1.31) | 3.45 (0.94) |  |
| ***Secondary Outcomes*** | | | | | | |  |
| **Cognitive and behavioral coping skills, mean (SD)** | | | | | | |  |
| Self-distraction | 2.98 (0.7) | 3.27 (0.70) | 3.04 (0.95) | 3.22 (0.69) | 2.92 (0.57) | 3.33 (0.70) |  |
| Active coping | 2.31 (0.66) | 2.47 (0.81) | 2.50 (0.68) | 2.45 (0.83) | 2.12 (0.62) | 2.49 (0.79) |  |
| Emotional support | 2.46 (0.89) | 2.35 (0.87) | 2.54 (0.97) | 2.28 (0.85) | 2.38 (0.85) | 2.41 (0.90) |  |
| Instrumental support | 2.27 (0.97) | 2.30 (0.90) | 2.50 (1.06) | 2.25 (0.84) | 2.04 (0.85) | 2.35 (0.96) |  |
| Venting | 2.83 (0.81) | 2.39 (0.82) | 3.00 (0.84) | 2.37 (0.80) | 2.65 (0.77) | 2.41 (0.84) |  |
| Positive reframing | 2.27 (0.79) | 2.22 (0.86) | 2.08 (0.89) | 2.23 (0.88) | 2.46 (0.66) | 2.20 (0.83) |  |
| Planning | 2.56 (0.90) | 2.58 (0.91) | 2.42 (1.04) | 2.58 (0.84) | 2.69 (0.75) | 2.58 (0.98) |  |
| Acceptance | 2.42 (0.78) | 2.82 (0.80) | 2.46 (0.78) | 2.83 (0.83) | 2.38 (0.82) | 2.82 (0.78) |  |
| Self-Blame | 3.23 (0.68) | 3.04 (0.92) | 3.23 (0.83) | 3.02 (0.88) | 3.23 (0.53) | 3.05 (0.96) |  |
| Substance Use | 1.17 (0.37) | 1.36 (0.78) | 1.27 (0.44) | 1.44 (0.83) | 1.08 (0.28) | 1.29 (0.72) |  |
| Behavioral disengagement | 2.46 (1.00) | 2.11 (0.85) | 2.58 (1.13) | 2.11 (0.79) | 2.35 (0.88) | 2.11 (0.90) |  |
| **Positive LGBTQ+ Identity, mean (SD)** | | | | | | |  |
| Authenticity | 5.23 (1.16) | 5.03 (1.40) | 5.05 (0.80) | 5.06 (1.43) | 5.42 (1.44) | 5.00 (1.38) |  |
| LGBTQ+ Community | 4.85 (1.38) | 4.81 (1.36) | 5.03 (1.51) | 4.93 (1.34) | 4.66 (1.28) | 4.69 (1.38) |  |
| **Internationalization of minority stress, mean (SD)** | | | | | | | |
| Internalization | 3.30 (1.00) | 3.24 (1.27) | 3.63 (0.72) | 3.16 (1.28) | 2.84 (1.17) | 3.33 (1.25) |  |
| **Sense of belonging, mean (SD)** | | | | | | |  |
| Thwarted belongingness | 19.38 (6.62) | 19.18 (6.18) | 20.31 (5.31) | 19.50 (6.22) | 18.46 (7.83) | 18.87 (6.14) |  |
| **Anxiety and depression symptoms, mean (SD)** | | | | | | |  |
| Anxiety | 12.62 (5.44) | 11.53 (5.36) | 12.69 (5.44) | 11.40 (5.30) | 12.54 (5.67) | 11.66 (5.43) |  |
| Depression | 12.73 (6.35) | 12.70 (5.58) | 11.69 (6.65) | 12.48 (5.91) | 13.77 (6.11) | 12.92 (5.24) | |

^a^ Anyone who identified as Transgender as one of their gender identities was classified as Transgender. Then, participants who identified as Non-binary, Agender, or Genderqueer as one of their gender identities were classified as Non-binary. Among rest of participants, those who identify Cisgender Man, Cisgender Woman, Man or Woman as one of their gender identities were classified as Cisgender, so long as their sex assigned at birth corresponded with their current gender identity. The rest of participants were classified as ‘Other’.

^b^ Anyone who identified as Bisexual or Pansexual as one of their sexual orientations were classified as Plurisexual. Then, participants who identified as Gay or Lesbian as their only sexual orientation were classified as Gay or Lesbian. The rest of participants were classified as Other.

^c^ Anyone who selected ‘They’ as one of their pronouns were classified as ‘They/They+’. Among the rest of participants, those who select ‘He’ or ‘She’ as one of their pronouns were classified as ‘He/He+’ or ‘She/She+’. The rest of participants were classified as ‘Other’.
